# The Expanding Truffle Environment: A Study of the Microbial Dynamics in the Old Productive Site and the New *Tuber magnatum* Picco Habitat

**DOI:** 10.3390/jof10110800

**Published:** 2024-11-19

**Authors:** Mara Rondolini, Maurizio Zotti, Gilberto Bragato, Leonardo Baciarelli Falini, Lara Reale, Domizia Donnini

**Affiliations:** 1 Department of Agricultural, Food and Environmental Sciences, University of Perugia, 06121 Perugia, Italy; 2 Department of Agricultural Sciences, University of Naples Federico II, Via Università, 100, 80055 Portici, Italy; 3 Research Centre on Viticulture and Enology, Council for Agricultural Research and Economics, 34170 Gorizia, Italy

**Keywords:** white truffles, soil, amplicon sequencing, fungi, prokaryotes, ecotone

## Abstract

Truffles are valuable underground mushrooms with significant economic importance. In recent years, their cultivation has achieved satisfactory results, but not for all species. The harvesting of white truffles (*Tuber magnatum* Picco) is still dependent on natural production, which is at risk due to various issues, such as improper forest management. A useful practice to protect natural resources is to promote the expansion of productive forests. In this study, we investigate the dynamics of the microbiome in an old and new truffle forest using an amplicon sequencing approach of the fungal ITS region and the prokaryotic 16S rRNA gene. We monitor the soil biological community’s development to compare differences and similarities between the primary productive forest and the expanding area over a two-year sampling period. In particular, we observed the colonization of vacant ecological niches by certain fungi, such as those belonging to the genus *Mortierella*. Additionally, we examined the competitive interactions between saprotrophs and ectomycorrhizal fungi (ECM). In both study areas, the bacterial community was dominated by Pseudomonadota, Planctomycetota, and Actinomycetota. The behavior of the *Tuber* genus differed significantly from other ECMs and displayed positive correlations with bacterial taxa such as *Ktedonobacter*, *Zavarzinella*, and *Sphingomonas*. The present work provides an initial overview of expanding white truffle habitats. Further, more specific research is needed to explore potential connections between individual *taxa*.

## 1. Introduction

The white truffle *Tuber magnatum* Picco is a hypogeous mycorrhizal fungus. The life cycle of the fungus relies on the formation of symbiotic relationships with plants in the form of ectomycorrhizas [1]. Few plant partners are able to carry out the mutualistic symbiosis and depend on coevolution and niche sharing [2]. However, the truffle’s entire biological cycle and reproduction are influenced by various factors, including soil characteristics and climate [3,4,5]. Particularly, the interaction of environmental variables, such as moisture, temperature, vegetation cover, soil structure and composition, and microbial communities [6], is crucial for the optimal development of ascocarps. 

The truffle holds significant economic value [1], leading to potential development opportunities in rural areas [7,8,9]. White truffles thrive in environments where other crops struggle, such as valley floors. This makes them a valuable resource for preserving local communities and attracting young people to rural life. Therefore, it is crucial to study and conserve these environments, which are highly vulnerable to issues related to climate change and land abandonment.

Although the production of mycorrhized plants with white truffles is challenging, it is possible to find good-quality plants in Italy and France. In spite of this, its cultivation has not yet yielded satisfactory results, unlike other truffles, so there is a growing interest in studying and conserving it in the natural environment [10,11,12].

Studies have been conducted on management techniques and ecological requirements [13,14]. In recent years, attention has been focused on the interaction with other soil organisms, especially bacteria, which have been defined as a third partner in the symbiosis between the fungus and the plant root [15]. Numerous studies have been conducted in the identification of bacterial communities associated with truffle ascocarps [16,17]. This discovery has highlighted their significant role in producing aromatic compounds [18,19] and their involvement in nutrient cycling [20].

The advent of next-generation sequencing (NGS) techniques allows investigation of the microbiome associated with truffle-producing soil and ascocarps [21]. The descriptions present in the literature offer valuable insights into the possible relationship between soil biological communities and truffles [22,23,24,25,26,27]. Recent studies have focused on the microbiome of the white truffle and its surrounding environment [21,28,29]. In vitro experiments have shown a positive interaction between the growth of *Tuber magnatum* mycelium and certain microorganisms, including *Bradyrhizobium* species [30]. However, comparative descriptions of the associated microbiome during the development of production habitats are lacking. Woodland dynamics as young, non-productive, or recently productive truffle beds may constitute an important reservoir of helpful information for white truffle sustainable exploitation as it constitutes a valuable ecosystem service due to the use of the fungus in gourmet cuisine. Normally the natural producing environments are those closest to new truffle plantations, realized in proximity with the attempt to find the ideal conditions for the development of a new truffle bed. 

In our research, we have concentrated on the ecotone, which is the transitional area of the forest. This is where the young seedlings of symbiotic trees create an optimal environment for the mycelial network to grow and, within a few years, gain the ability to produce ascocarps. We described the soil biological communities in this transitional environment between the productive forest and the agricultural field, in synchronic and diachronic ways, with the aim to: (i) identify the most representative *taxa* of the truffle forest and the ecotone, highlighting differences; (ii) observe how the community changes over time and determine if the dynamics of the microbiome are consistent between both areas.

## 2. Materials and Methods

### 2.1. Study Area

**Figure 1:**
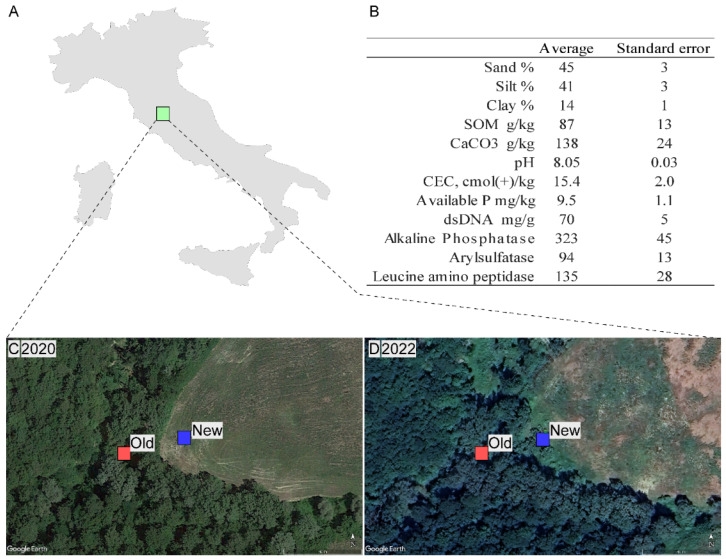
(**A**) Location of experimental site in Italy (43°9′20″52 N 11°35′27″24 E); (**B**) Description of soil characteristics of the study site; satellite images of the study sites in 2020 (**C**) and in 2022 (**D**) with respective *Old* (red squares) and *New* (blue squares) sampling points.

This study was conducted in San Giovanni d’Asso, a location in southern Tuscany, Italy (43°9′20″52 N 11°35′27″24 E). The area is famous for its unique hillside landscapes, which are characterized by gullies: forms of slope erosion that suggest a discrete sandy component in the soil. The valleys at the base of these hills collect eroded sediments and rainfall from the slopes, defining an environment with deep, porous soils, perfect characteristics for developing white truffles [31]. The area under study was located in one of these valley bottoms, where the owner has been managing the forest for years to conserve and improve the truffle resource and where he is promoting its natural expansion bordering an abandoned cultivated field. The actions implemented are to cut and reduce the vegetation around poplar seedlings (*Populus canescens* (Aiton) Sm.), the main symbiont plant in the area, and provide water if necessary, promoting their growth and development. As already mentioned, soil texture is loam, with 45% sand and 14% clay. Other soil characteristics are reported in Figure 1.

### 2.2. Experimental Design, Soil Sampling, and DNA Extraction

We designed the experiment as a comparison between producing woodland (*Old*) and the ecotonal area with expanding renewal of potential symbionts (*New*). Soil samples were collected in the autumn of the years 2020 and 2022. Six samples were collected in the expanding area, which we will refer to as “*New*”, and another six in the adjacent truffle forest, which we will refer to as “*Old*”. Core samples were collected using a previously sterilized, 16 mm diameter PVC tube. The tube was hammered into the soil to a depth of 20 cm after removing the litter layer. A total of 24 samples were collected in two sites and two productive seasons. The cores were transported to the laboratory with a portable refrigerator at 4 °C using ice packs and then stored at −80 °C. Before molecular analysis, the samples were freeze-dried and then pounded. Samples were sieved at 500 µm to exclude stones and root debris. The whole procedure was carried out taking care to disinfect each instrument before processing another sample. DNA was extracted from about 0.40 gr of soil per sample using a Qiagen, Hilden, Germany, DNeasy PowerSoil Kit (Cat No./ID: 12888-100) following the manufacturer’s instructions. The DNA was eluted with 50 μL of distilled water and stored at −20 °C until further analysis.

### 2.3. Bioinformatic Analyses

PCR analyses were conducted by Sequentia Biotech SL (Barcelona, Spain), including library preparations, Illumina MiSeq sequencing of the bacterial 16S rRNA genes and fungal ITS region, and bioinformatic analysis.

The primers used for amplification of the 16S region are 341F 5′-CCTACGGGNGGCWGCAG-3′ and 805R 5′-GACTACHVGGGTATCTAATCC-3 and for the ITS region they are ITS1 5′-TCCGTAGGTGAACCTGCGG-3′ and ITS2 5′-GCTGCGTTCTTCATCGATGC-3′. Library generation was carried out following the protocol recommended by the kit manufacturer (Illumina, San Diego, CA, USA). The raw data were subjected to a quality check using the software BBDuk. The trimming was performed by removing the low-quality parts of the data while keeping intact the longest high-quality part of the data. The minimum read length required for the analysis was set at 35 bp, with a quality score of 25 to ensure high-quality and reliable results. The software GAIA (version 2.02, Sequentia Biotech, Spain) was used to analyze the taxonomic profiling of the samples. The process involves aligning each set of reads with a reference database to extract the best alignments for accurate comparison, and then a lowest common ancestor (LCA) algorithm is applied to identify the best alignments. The analysis returned a table of OTUs for each taxonomic level. Raw sequence reads have been submitted to the Sequence Read Archive linked to the bio-project number PRJNA1167916 in the National Center of Biotechnology Information “https://www.ncbi.nlm.nih.gov/bioproject/ (accessed on 2 October 2024)”.

### 2.4. Statistical Methods 

Alpha diversity indices were calculated using the *vegan* package [32] with the “specnumber” function for the richness and “diversity” function for Shannon’s index calculation. Also, the number of reads for each sample was used as indicator of alpha diversity. Significative changes in species richness, read number, and Shannon index were tested by means of two-way analysis of variance (two-way ANOVA). 

Beta diversity between the samples was calculated with the Bray–Curtis dissimilarity index using the “vegdist” function of the *vegan* package. For both bacterial and fungal communities, the Bray–Curtis dissimilarity matrix was calculated and multidimensional ordination of samples was visualized by non-metric multidimensional Scaling (NMDS). Ordinations were performed with “metaMDS” and permutational analysis of variance (PERMANOVA) was used to quantify the impact of variables on dissimilarity between communities with the “adonis2” function. To detect the taxa-specific variations in bacterial and fungal communities, heatmaps were built comparing *New* and *Old* sites between different seasons for both bacterial and fungal communities, restricting the analysis to the 70 most representative *taxa*. To identify microbial groups according to their abundance in experimental design, variables were ordered using hierarchical clustering based on the index of association. Furthermore, volcano plots were drawn to isolate the main statistically significant *taxa* producing sample differentiations. Metrics for volcano plots were calculated as fold changes between years for *New* and *Old* sites and significance was calculated by means of a *t*-test for homoscedastic data.

The most abundant fungi in the different areas, classified at the genus level, were annotated with their trophic function via the FUNGuild database using the “funguild_assign” function in the *FUNGuildR* package [33]. FUNGuild data, frequency of *Tuber* genus, and the most representative bacteria highlighted by volcano plot were checked for correlation by means of Spearman rank values with the aim to understand microbiological dynamics and possible relationships in *Old* and *New* forested soils. The resulting correlation maps were ordinated for fungal trophic groups and bacteria by means of hierarchical clustering based on Euclidean distance metrics. Statistical analyses were carried out in the R environment (R Core Team 2020) and the Primer 7 software (PRIMER-E Ltd., Plymouth, UK).

## 3. Results

**Figure 2:**
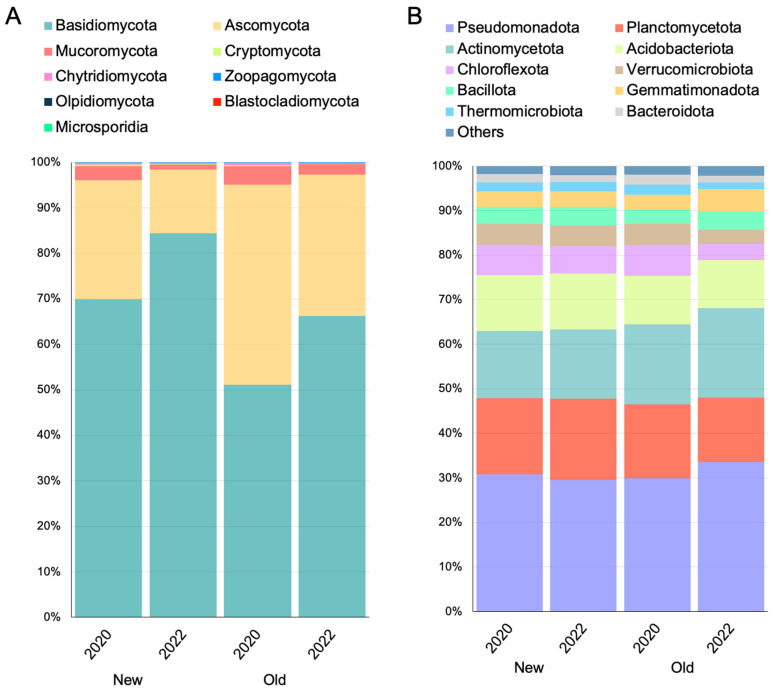
Stacked bar chart of the identified OTUs at the phylum level. (**A**) The percentage of detected fungal phyla; (**B**) The percentage of detected prokaryotic phyla.

Bioinformatic analysis returned 18431100 OTUs for bacteria and 10928616 OTUs for eukaryotes. Taxonomic analysis at the phylum level showed minimal differences in the bacterial community and was more marked for fungi (Figure 2). 

For the fungal community, members of Basidiomycota are the most representative *taxa*. Specifically in the *New* site, Basidiomycota are more abundant in the expanding area compared to the *Old* one (77.12% in *New* vs. 66.22% in *Old*). Additionally, a higher frequency of Basidiomycota was observed in 2022 for both experimental areas.

The second and the third most representative phyla were Ascomycota and Mucoromycota, respectively, which showed the same pattern of change in terms of frequency, with a sharp decline in 2022 for both *New* and *Old* areas. 

Pseudomonadota, Actinomycetota, and Planctomycetota are the predominant bacterial *taxa* in the study areas. The former showed a differential effect according to the year and the site was particularly favored in the *Old* area in 2022 (33.55%) while depressed in the *New* area of the same year. Actinomycetota are disadvantaged in the expanding area (~15% in *New* vs. ~19% in *Old*) but do not undergo great changes over the years within the same site. Instead, Planctomycetota showed a distinct association with the expanding area compared to the *Old* one (~17.5% in *New* vs. ~15% in *Old*).

### 3.1. Alpha and Beta Diversity

**Figure 3:**
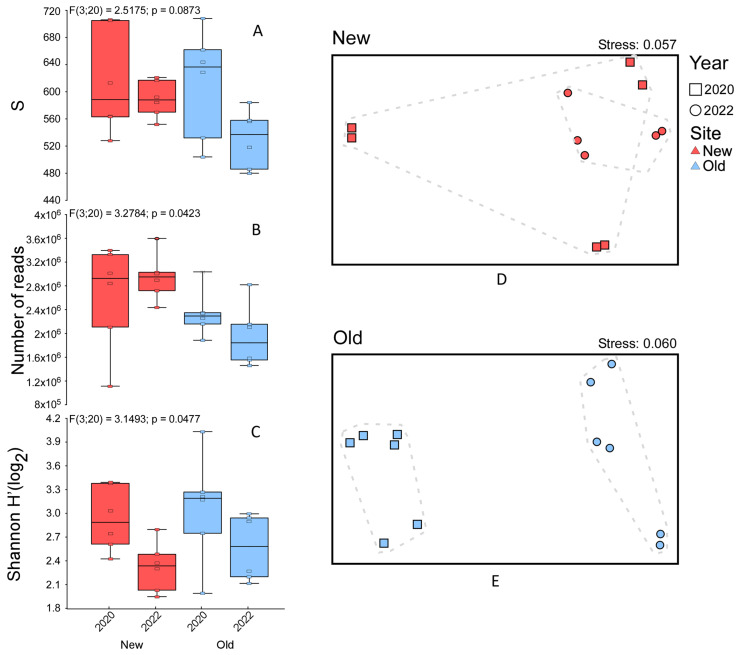
Alpha diversity boxplots showing species richness, number of reads, and Shannon index for the fungal community for each site (red for *New* and blue for *Old*) in different years (**A**–**C**). Non-metric multidimensional scaling (NMDS) plots and relative stress level, using Bray–Curtis dissimilarity matrices of fungal community for *New* site (**D**) and *Old* site (**E**).

The alpha diversity analysis for the fungal community showed no significant change in species richness between years and sampling areas (F = 2.51; *p* = 0.087), however, a notable decrease is observed in some samples from the *Old* area in 2022 (Figure 3A). Inversely, the number of reads significantly decreased in the *Old* area compared to the *New* one with no effect observed for the years (F = 3.27; *p* = 0.042; Figure 3B). Shannon index values also change significantly, showing a dominant pattern marked by lower values in *New* and *Old* areas in 2022 (F = 3.14; *p* = 0.047; Figure 3C). NMDS of the *New* area showed that the fungal community does not differ between years. However, the fungal community appears more similar between sampling points in 2022 than between sampling points in 2020 (PERMANOVA, PER = 999, *p* = 0.019; Figure 3D). The same ordination for samples of the *Old* area instead shows a clear differentiation in the fungal community between years (PERMANOVA with 999 perm. *p* = 0.002; Figure 3E).

**Figure 4:**
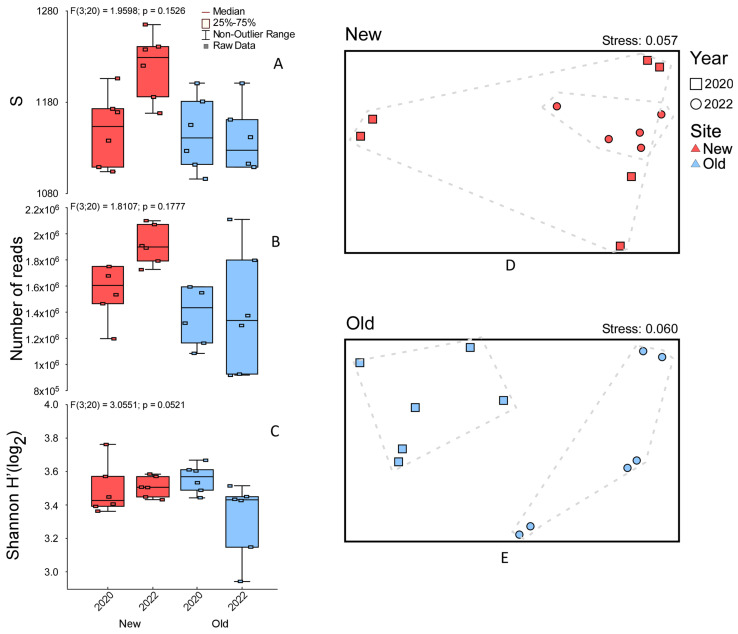
Alpha diversity boxplots showing species richness, number of reads, and Shannon index for the prokaryotic community for each site (red for *New* and blue for *Old*) in different years (**A**–**C**). Non-metric multidimensional scaling (NMDS) plots and relative stress level, using Bray–Curtis dissimilarity matrices of fungal community for *New* site (**D**) and *Old* site (**E**).

For bacterial community, alpha diversity metrics show no significant differences between samples. However, an increase in bacterial richness and number of reads is observed in the *New* area in 2022 (Figure 4A–C). In the *New* area, NMDS showed overlap of community similarities between 2020 and 2022 but with a marked clustering of 2022 sites (PERMANOVA with 999 perm., *p* = 0.067; Figure 4D). In the *Old* area, as also observed for fungi, clear differentiation of the bacterial community occurs between 2020 and 2022 (PERMANOVA with 999 perm., *p* = 0.004; Figure 4E).

### 3.2. Microbiome Associated with the Expanding Truffle Habitat 

**Figure 5:**
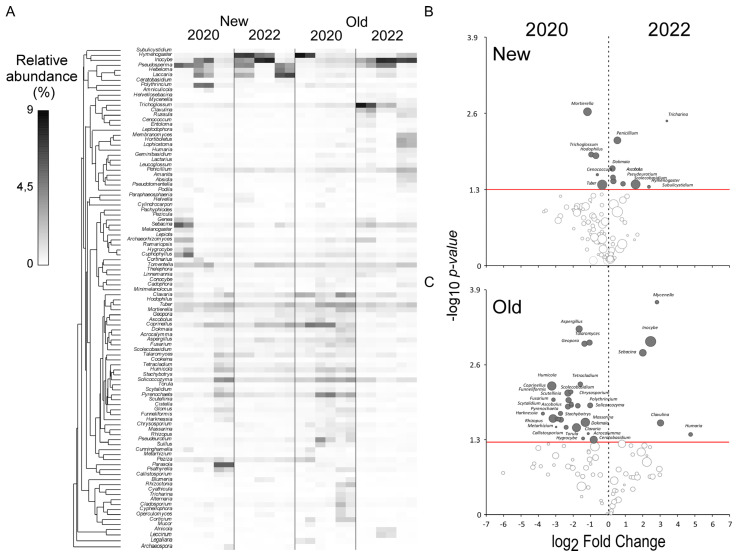
(**A**) Heatmap showing the relative abundance of the 70 most representative fungal *taxa* for each site in different years. Hierarchical clustering is based on index of association; the volcano plots show the patterns of enrichment and diminishment in the fungal community through the years in the *New* site (**B**) and the *Old* site (**C**). The dark dots indicate a significant increase on the right and a significant decrease on the left, while empty dots represent *taxa* with no significant difference in abundance.

**Figure 6:**
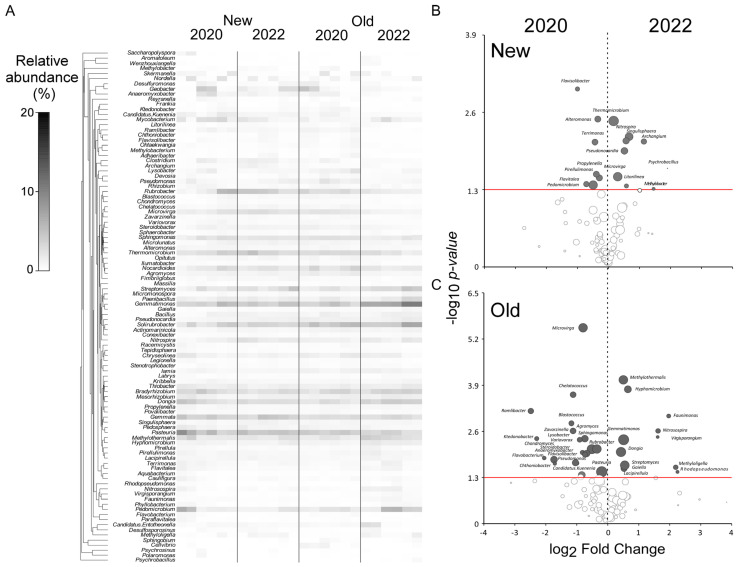
(**A**) Heatmap showing the relative abundance of the 70 most representative prokaryotic *taxa* for each site in different years. Hierarchical clustering is based on index of association; the volcano plots show the patterns of enrichment and diminishment in the prokaryotic community through the years in the *New* site (**B**) and in the *Old* site (**C**). The dark dots indicate a significant increase on the right and a significant decrease on the left, while empty dots represent *taxa* with no significant difference in abundance.

*Taxa* contributing to differentiation of sampling areas are shown in heatmaps and volcano plots of both fungal (Figure 5) and bacterial (Figure 6) communities. 

Among fungi, the *New* area is differentiated because of the high frequency of *taxa* belonging to *Tuber*, *Cenococcum*, *Hodophilus*, *Trichoglossum*, and *Mortierella* in 2020 (Figure 5B). In 2022 those genera were significantly substituted *by Subulycistidium*, *Hymenogaster*, *Scolecobasidium*, *Pseudeurotium*, *Ascobolus*, *Dokmania*, *Penicillium*, and *Tricharina*. Also worth mentioning, although not significant, is the 2022 increase in *Inocybe*, *Laccaria*, *Penicillum*, and *Coprinellus* (Figure 5A). In 2020 the *Old* area was characterized by a higher frequency of *Aspergillus*, *Talaromyces*, *Geopora*, *Humicola*, *Tetracladium*, and *Coprinellus*. The fungi of the genus *Tuber* also showed an increase, although not significant, in relative abundance in 2020. In 2022 the community shift was mainly characterized by the higher abundance of *Inocybe*, *Mycenella*, *Sebacina*, *Clavulina*, and *Humaria* (Figure 5C). Notably, fungi of the genus *Pseudosperma* also show a tendence to increase in relative abundance in 2022. 

In the *New* area the bacterial community between different years changed because of the high presence of *Flavisolibacter*, *Alteromonas*, *Terrimonas*, *Propylenella*, *Pirellulomonas*, *Flavitalea*, and *Pedomicrobium* in 2020. The bacterial composition of soil shifted in 2022 towards a community mainly composed of *Thermomicrobium*, *Nitrospira*, *Singulisphaera*, *Archangium*, *Pseudonocardia*, *Microvirga*, *Litorilinea*, and *Methylobacter* (Figure 6B). In the *Old* habitat the change between years was mainly due to a high frequency of *Microvirga*, *Chelatococcus*, *Ramilibacter*, *Blastococcus*, *Abromyces Sphingomonas*, *Rubromyces*, and *Pasteuria*, whereas *Methylothermalis*, *Hyphomicrobium*, *Gemmatiomonas*, *Dongia*, and *Streptomyces* were favored in 2022 (Figure 6C). 

### 3.3. Ecological Dynamics of Mycobiome and Associated Bacterial Consortia 

The stacked bar plot of FUNGuild data showed different fungal community patterns when grouped according to their trophic strategy (Figure S1). The differentiation is mainly due to the different years of sampling, as in 2022 a dominance of ECM fungi was recorded for both the *New* and *Old* areas. When comparing the two years for each area, it can be noticed that in the *New* site the dominance of ECM excludes many saprobic *taxa*. It also excludes plant pathogens, epiphytes, and, to a lesser extent, animal parasites. The same happens in the *Old* habitat, where in 2022 the dominance of ECM becomes disadvantageous for plant pathogens, saprotrophs, epiphytes, and other minor fungal groups.

**Figure 7:**
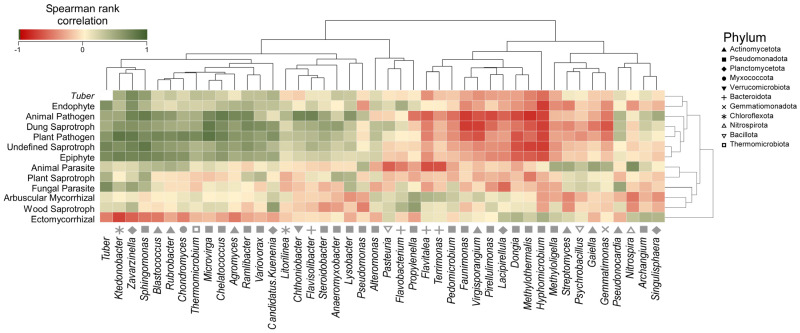
Spearman’s rank correlation maps showing the relationships between *Tuber* genus, fungal trophic groups, and bacteria. Hierarchical clustering is based on Euclidean distance metrics.

Accordingly, when clustering fungal guilds together (Figure 7), Euclidean distance values showed that endophytes and fungi belonging to the *Tuber* genus have similar patterns in forest soil (Cluster 1). While animal pathogens, saprotrophs, plant pathogens, and epiphytes clustered together (Cluster 2), other groups such as animal parasites, plant saprotrophs, fungal parasites, arbuscular mycorrhizal fungi, and wood saprotrophs showed no strong associative patterns between each other (Cluster 3). ECM groups showed a completely opposite effect compared to the other fungal groups (Cluster 4). When defining bacterial consortia associated with fungal trophic groups, it can be observed that Cluster 1 is associated with bacteria of the *Ktedonobacter*, *Zavarzinella*, and *Sphingomonas* genera, whereas Cluster 2 is associated with *Blastococcus*, *Rubrobacter*, *Chondromyces*, *Thermomicrobium*, *Microvirga*, and others. Cluster 3 showed no correlation with microbial groups, while ECM fungi (Cluster 4) positively correlated with *Lacipirellula*, *Dongia*, *Hyphomicrobium*, *Singulisphaera*, and *Archangium* genera. 

## 4. Discussion

In recent times, several studies have focused on the relationship between truffles and the environmental microbiome to understand some key associations and gain insight into the biology of those species [19,21,29,34,35,36]. For example, Vahdatzadeh et al. (2015) [37] demonstrated that the valuable aromatic characteristic of truffles derives from the association of bacteria. Lalli et al. (2015) [38] noted an affinity of *Amanita stenospora* Contu, *Cortinarius aprinus* Melot, *Hebeloma quercetorum* Quadr., and *Hygrophorus arbustivus* Fr. var. *quercetorum* Bon & Chevassut for the white truffle habitat, while *Mortierella* and *Fusarium* were found to be abundant in truffle soil by Mello et al. (2010) [39]. In the present study, we investigated the natural habitat dynamics in a truffle forest and its expansion into a nearby abandoned field that took place from 2020 to 2022.

### 4.1. Fungal Dynamics in the Expanding Truffle Forest

The most notable findings of our survey on the fungal community at the phylum level highlight an increase in the relative abundance of Basidiomycetes reads. This shift may be due to rainfall and temperature variations in 2022, which likely created more favorable conditions for the mycelial expansion of this phylum. Accordingly, the dominance of Basidiomycota in 2022 is coupled with the overall increase in dominance suggested by alpha diversity and the decrease in evenness among fungal *taxa*, which means that few *taxa* were proliferating in the soil environment as a result of out-competition with the previous community. The impact of dominant Basidiomycete mycelial mats has been well-documented, especially in processes related to organic matter decomposition and within grassland ecosystems [40,41,42,43]. Basidiomycetes often play a key role in the later stages of organic matter decomposition and habitat colonization [44,45], as they are more effective in the degradation of recalcitrant molecules such as lignin, celluloses, and phenols because of the wider enzymatic arsenal and oxidative trophic strategies [46,47]. Many fungi within this group are known to monopolize resources and actively defend them from potential competitors [48,49].

When considering the internal variability in the *New* and *Old* forests, we observed a higher fungal diversity in the *New* area, with a net distinction in community composition within the sampling group. This indicates that in the early stage of colonization of a potential forest niche, fungal communities can assume different shapes, with a heterogeneous community composition [40]. This probably depends on the first arrival and establishment in the new empty niches, that is unpredictable in terms of *taxa* composition [50]. With the establishment of a forest environment over time, the fungal community stabilizes and homogenizes its composition in parallel with plant cover and species normalization [51,52]. Interestingly, the *New* expanding area in this study was managed by the owner to favor potential plant hosts for *T. magnatum* colonization. The results observed may be due to this selective practice [51]. Remarkably, the alpha diversity indices confirm the high variability of the *New* expanding area and outline that in the ecotone fungal species richness and number of reads tend to display higher values compared to the *Old* area. The latter is, instead, characterized by lower variability because of the longer time that passed for fungal community establishment since habitat formation. Hence, the higher diversity in the *New* area may be due to the opportunistic colonization by many ruderal species, which is a general rule in the ecology of colonization of a new environment at the multikingdom level [42,53]. When considering the change in the *New* area at a higher taxonomic level (genus), the specific changes were not clearly visible as higher variability found in 2020 decreased the number of *taxa* with significant frequency shifts. However, fungi of the genus *Mortierella* provide a clear signal of opportunistic colonization of empty niches, as already found in many disturbed or non-structured soil environments [54]. In 2022, on the other hand, the significative expansion of *Hymenogaster*, an ECM-forming fungus [55,56], is in accordance with the observation regarding the dominance of the Basidiomycota at the phylum level. Interestingly, the production season of 2020 was particularly good (personal communication from the study area owners), coinciding with a high frequency of Tuber spp. reads. In the *Old* area, the stabilization of the community throughout the life cycle of the habitat results in a clearer representation of community change over the years. In 2020 the community was composed of a high variety of fungal *taxa* with different trophic guilds, quickly replaced by a poorer community that is, instead, dominated by the mycelia of ECM species, mostly of the *Inocybe*, *Humaria*, *Sebacina*, and *Clavulina* genera [6,55,57]. Those observations at the genus level are also confirmed by an analysis at the guild level that outlines an important enrichment of ECM mycelium in soil in the second sampling year. When environmental conditions became favorable for ECM mycelial exploration of soil volume, the exclusion of many saprobic species in a process called the Gadgil effect takes place [58,59]. The competition between the two fungal guilds for organic nutrients and other soil resources is believed to result in the deceleration of the decomposition rate of organic matter (SOM), favoring the ECM lifestyle. In addition, the ecological niches of the two groups are usually separated. Saprophytic fungi are typically found in the superficial part of the soil, where there is a higher amount of SOM, while ECMs are generally located at greater depths, except for a few species [60,61]. In our study, samples were collected at a depth of 20 cm, which may have favored the observation of more ECMs than saprotrophs. Furthermore, the landowner’s management interventions in 2022 may have impacted the dynamics of the communities. Specifically, the area is involved in periodically cutting the excess shrub vegetation to promote continuous forest rejuvenation. According to this intervention, the ecotone has also undergone a selective cutting that favors the growth of young symbiotic plants. The removal of most of the plant residues may also result in a lower amount of decomposable litter, thus favoring the presence of ECM fungi.

### 4.2. Bacterial Dynamics in the Expanding Truffle Forest

In the prokaryotic community, we observed a certain level of stability in the dominant *taxa*, which include Pseudomonadota, Planctomycetota, and Actinomycetota. However, bacterial dynamics partially mirrored what was observed in the analysis of the fungal community, in beta diversity in particular. This leads us to think that the few changes observed might be linked to the expansion of the mycelium of certain *taxa* of the fungal guilds in soil. In recent years attention on the relationships between bacterial *taxa* and fungal mycelium increased, facilitated by the advent of NGS [62,63]. The changes we observed indicate that Pseudomonadota (Proteobacteria) is the bacterial phylum that predominates in the study areas, in particular in the *Old* area in 2022. Bacterial *taxa* are normally associated with the release of nutrients in the environment as many of those species are copiotrophics, with a strong dependence on nitrogen budget [41,64]. It may be argued that in the period of stronger mycelial activity (2022) the decomposition of organic matter carried out by dominant ECM community may release nutrients in a simplified way. In fact, in the area with a higher frequency of Pseudomonadota, members of Hyphomycrobiales thrive, including bacteria belonging to *Methilobacter*, *Dongia*, *Hyphomicrobium*, *Archangium*, and *Methilotermalis* genera. Interestingly, these *taxa* showed a positive correlation with the abundance of ECM species in soil. On the other hand, the high amount of fungal mycelium and its senescent portion may release high amounts of nutrients in the soil environment [42,64]. The relationship with the ability of ECM species to decompose SOM was further suggested by the presence of bacterial genera specialized in the sequestration of P like *Gemmatimonas* [65] and the acidophilic planctomycete *Singulisphaera* [66]. However, among the bacterial *taxa* that are significantly enriched, many Proteobacteria have shown a negative correlation with many other fungal guilds. This suggests that the association between fungi and bacteria may be more appropriately studied by considering species-specific interactions. However, the actual data provided in our study do not allow us to go into more depth on the possible interactions occurring between the two kingdoms, requiring specific experimental data to confirm possible hypotheses. 

### 4.3. Microbial Relationships of Truffle Mycelium in Expanding Truffle Forests

The Spearman rank correlation in Figure 7 illustrates an inverse relationship between the two fungal guilds. Even though the *Tuber* genus is part of the ECMs, we observe a different trend compared to its guild. This could have two explanations, both related to the *Tuber* biological cycle. Firstly, the mycelium growth rate appears to be highest in spring, then decreases significantly in summer, and starts to increase again in autumn, when it concentrates on producing ascocarps [67]. In this study, soil sampling was performed in autumn at random points in each area. The aim was to describe the soil environment regardless of where exactly white truffle ascocarps are located. This might explain the lower presence of the *taxon* in question. However, this could also lead to the hypothesis of different conservation strategies: for example, organisms might move to greater depths in search of subsoil water resources and less competition [68]. Another strategy could be the association with generally non-host plants, assuming an endophytic lifestyle as demonstrated for other *Tuber* species [69], making it difficult to detect the *Tuber* genus. This second hypothesis could also explain the positive correlation found with endophytes (Cluster 1, Figure 7) that, along with the other associations, needs further investigation. Concerning the specific correlation of *Tuber* mycelium with bacterial *taxa*, the former presents a high association with *Ktedonobacter*, *Zavarzinella*, and *Sphingomonas* members of *Chloroflexota*, *Plantomycetota*, and *Pseudomonadota*, respectively. For the first two, no interaction with *Tuber* spp. has been described in the literature. The first genus has a wide ecological range, proliferating in both common and extreme environments [70], while the second is a monospecific genus (*Zavarzinella formosa*) recently described as a new species and isolated in peat from a boreal environment [71]. On the contrary, the *Sphingomonas* genus was found in soil (roots) truffle sites and ascomata of *T. aestivum* Vittad. [24,26]. These bacteria are able to enhance plant growth and drought resistance through multiple mechanisms, for instance, stimulating the formation of secondary roots [72] essential for the formation of new mycorrhizas [73,74,75]. The species *Sphingomonas wittichii* was also found to co-occur with *Tuber melanosporum* Vittad. [76]. Pavić et al. in 2011 [77] isolated *Sphingobium* sp. TMG 022C from an ascocarp of *T. magnatum* and demonstrated its ability to perform ammonification and nitrate reduction, solubilize phosphate, hydrolyze lipids, and degrade β-glucans and chitin, suggesting that it could be involved in mycelium nutrition and ascocarp growth and decomposition.

## 5. Conclusions

In this study, we presented a description of the truffle soil microbiome in a productive forest and a nearby expanding area. The extension of truffle forests could be a valid management technique for the preservation of the resource in nature. It is important to assess each individual environmental situation in order to identify the specific methods of intervention. In the study area, expansion is stimulated by selective cutting, leaving the seedlings of the symbionts, and cutting the rest until the young plants acquire vigor. The results indicate a balanced biological community in the primary forest, which seems to be particularly sensitive to external factors, as evidenced by the differences found between the two sampling years. In the expanding truffle forest, we find bacterial and fungal communities that are not yet well-defined and have greater dynamism in response to environmental changes. Another interesting interaction was observed in the competition between saprophytes and ECMs. The behavior and dynamics of the latter in response to forest management interventions in the study area show a preference for litter-limited conditions. Looking closely at the dynamics of the fungi of the *Tuber* genus, we found significant differences in the trophic guilds they belong to. This emphasizes that the interactions between individual *taxa* are influenced by their biology, therefore suggesting the need for more research to understand the relationships between the specific *taxa* in the environment.

## Data Availability

Raw sequence reads have been submitted to the Sequence Read Archive linked to the bio-project number PRJNA1167916 in the National Center of Biotechnology Information “https://www.ncbi.nlm.nih.gov/bioproject/ (accessed on 2 October 2024)”.

## Supplementary Materials

The following supporting information can be downloaded at: https://www.mdpi.com/article/10.3390/jof10110800/s1, Figure S1: Stacked bar plot showing the different patterns in relative abundance of fungal data based on FunGuild trophic strategy associations.
