# Supplementary figures and images for: The Expanding Truffle Environment: A Study of the Microbial Dynamics in the Old Productive Site and the New Tuber magnatum Picco Habitat
Source: J Fungi (Basel). 2024 Nov 19;10(11):800. doi: 10.3390/jof10110800 (PMC11595706; doi:10.3390/jof10110800)

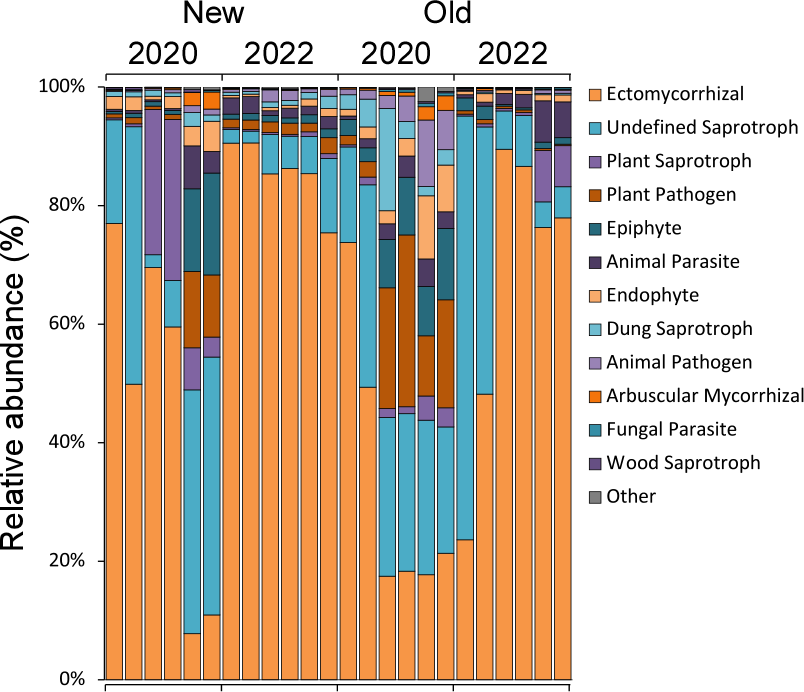

Supplement: Supplementary file 1 [file jof-10-00800-s001.zip › FigureS1.tif]
